# The platelet-derived growth factor receptor/STAT3 signaling pathway regulates the phenotypic transition of corpus cavernosum smooth muscle in rats

**DOI:** 10.1371/journal.pone.0172191

**Published:** 2017-02-28

**Authors:** Jun-Feng Yan, Wen-Jie Huang, Jian-Feng Zhao, Hui-Ying Fu, Gao-Yue Zhang, Xiao-Jun Huang, Bo-Dong Lv

**Affiliations:** 1 The Second Clinical Medical College, Zhejiang Chinese Medical University, Hangzhou, China; 2 Department of Urology, The Second Affiliated Hospital, Zhejiang Chinese Medical University, Hangzhou, China; 3 Andrology Laboratory on Integration of Chinese and Western Medicine, Zhejiang Provincial Key Laboratory of Traditional Chinese Medicine, Hangzhou, China; 4 Central Laboratory, The Second Clinical Medical College, Zhejiang Chinese Medical University, Hangzhou, China; Qatar University College of Health Sciences, QATAR

## Abstract

Erectile dysfunction (ED) is a common clinical disease that is difficult to treat. We previously found that hypoxia modulates the phenotype of primary corpus cavernosum smooth muscle cells (CCSMCs) in rats, but the underlying molecular mechanism is still unknown. Platelet-derived growth factor receptor (PDGFR)-related signaling pathways are correlated with cell phenotypic transition, but research has been focused more on vascular smooth muscle and tracheal smooth muscle and less on CCSMCs. Here, we investigated the role of PDGFR-related signaling pathways in penile CCSMCs, which were successfully isolated from rats and cultured *in vitro*. PDGF-BB at 5, 10, or 20 ng/ml altered CCSMC morphology from the original elongated, spindle shape to a broader shape and promoted the synthetic phenotype and expression of the related proteins vimentin and collagen-I, while inhibiting the contractile phenotype and expression of the related proteins smooth muscle (SM) α-actin (α-SMA) and desmin. Inhibition of PDGFR activity via siRNA or the PDGFR inhibitor crenolanib inhibited vimentin and collagen-I expression, increased α-SMA and desmin expression, and considerably inhibited serine-threonine protein kinase (AKT) and signal transducer and activator of transcription 3 (STAT3) phosphorylation. STAT3 knockdown promoted the contractile phenotype, inhibited vimentin and collagen-I expression, and increased α-SMA and desmin expression, whereas AKT knockdown did not affect phenotype-associated proteins. STAT3 overexpression in CCSMC cells weakened the suppressive effect of PDGFR inhibition on the morphology and phenotypic transformation induced by PDGF-BB. Through activation of the PDGFR/STAT3 signaling pathway, PDGF promoted the synthetic phenotype transition; thus, regulation of this pathway might contribute to ED therapy.

## Introduction

Erectile dysfunction (ED) is a common clinical disease that can affect the quality of life of patients and even the harmony and stability of their families [1]. Corpus cavernosum smooth muscle cells (CCSMCs) are known to be the main effector cells in the male erectile response and comprise 42–50% of the cells in the corpora cavernosum [2]. As the penis is considered as a vascular tissue in a specialized configuration [3], many changes in the cardiovascular system are expected be found in the penis. In contrast to the skeletal muscle cells and myocardium, which are in a terminally differentiated state, vascular smooth muscle cells (VSMCs) retain extensive plasticity, allowing them to undergo phenotypic modulation from a contractile state to a synthetic state in response to local environmental stimuli [4]. Phenotypic modulation is characterized by increased proliferation, migration, extracellular matrix production, and vimentin and collagen-I expression combined with decreased expression of contractile cytoskeletal proteins such as smooth muscle (SM) α-actin (α-SMA), SM myosin heavy chain (SMMHC), calponin, and desmin [5]. For approximately 23 h of each day, the penis of most adult men is in the flaccid state; thus, CCSMCs spend most of their time in the contracted state and retain a contractive phenotype [6,7]. Our previous studies have confirmed that CCSMCs possess the ability to modulate their phenotype from a contractile to a synthetic state under hypoxic condition, and these changes could play a role in cavernous nerve injury-induced ED in rats [8,9].

Platelet-derived growth factor (PDGF) is the most robust phenotype-modulating agent and comprises a family of heterodimers encoded by four genes PDGF-A, PDGF-B, PDGF-C, PDGF-D. PDGF acts on cells by binding to homo- or heterodimers of the two PDGF receptor proteins, PDGFRα and PDGFRβ [10,11]. Among the members of the PDGF family, only PDGF-BB can bind both to PDGFRα and PDGFRβ; therefore, it is more important than other isoforms in the pathogenesis of a variety of diseases [12]. Increasing data demonstrate that PDGFR-related signaling pathways are correlated with cell phenotypic transition. PDGFR interacts with PDGF to facilitate cell mitosis [13,14], which has a close relationship with processes such as growth and development, wound healing, atherosclerosis, and tumorigenesis [15,16]. PDGF induces the proliferation and phenotype change of airway smooth muscle cells [17]. Moreover, the PDGFR pathway regulates the vascular smooth muscle phenotype via mammalian target of rapamycin complex 1 and also contributes to attaining the synthetic phenotype under high oxidative stress in vascular lesions [18]. PDGF is expressed at high levels in the penile tunica albuginea of patients with Peyronie’s disease, suggesting that it could be involved in the pathogenesis of tunica albuginea fibrosis, which is frequently associated with ED in men [19]. In addition, PDGF infusion promotes the proliferation and migration of CCSMCs and causes phenotypic transition from the contractile to synthetic type [20]. However, to our knowledge, the regulatory mechanism of the PDGF/PDGFR pathway in CCSMCs is poorly understood.

In this study, we focused on the relationship between the PDGFR signaling pathway and the phenotypic transition of CCSMCs and attempted to identify the mechanism regulating the transition, which might aid in clinical therapy of ED.

## Material and methods

Crenolanib (CP-868596) was purchased from Selleck Chemicals LLC (USA). Recombinant human PDGF-BB protein was purchased from R&D system (USA). α-SMA, desmin, vimentin, and collagen-I, signal transducer and activator of transcription 3 (STAT3), phosphor-STAT3 (p-STAT3), signal transducer and activator of transcription 5 (STAT5), phosphor-STAT5 (p-STAT5), serine-threonine protein kinase (AKT), phosphor-AKT (p-AKT), extracellular regulated kinase (ERK), phosphor-ERK (p-ERK), PDGFRα, phosphor-PDGFRα (p-PDGFRα), PDGFRβ, phosphor-PDGFRβ (p-PDGFRβ), and β-actin were purchased from Abcam technology (USA).

### Cell isolation and culture

In total, 10 adult male Sprague-Dawley rats aged 8 weeks (body weight: 275–325 g) were purchased from SLRC Laboratory Animals (Shanghai, China). All animal experiments were performed in strict accordance with the recommendations of the Guide for the Care and Use of Laboratory Animals of the National Institutes of Health. The protocol was approved by the Committee on the Ethics of Animal Experiments of Zhejiang Chinese Medical University (permit number 2008–0115). All of the surgical procedures were performed under anesthesia induced by sodium pentobarbital administration, and all efforts were made to minimize suffering.

The primary culture of CCSMCs obtained from Sprague-Dawley rats was prepared as described in a previous study [21]. Briefly, the corpus cavernosum tissues from rats were washed with ice-cold phosphate-buffered saline (PBS) several times and cut into 1- to 2-mm^3^-thick segments. The segments were incubated in 0.5% collagenase A for 4 h and cultured at 37°C in a humidified atmosphere of 95% air and 5% CO_2_. Then, the cavernosal tissues were placed uniformly at the bottom of a 25-cm^2^ culture flask (Corning, NY, USA) containing approximately 2 ml of high-glucose Dulbecco’s modified Eagle medium (DMEM; Invitrogen, Grand Island, NY, USA) supplemented with 20% fetal bovine serum (FBS; Invitrogen), 100 U/ml penicillin, and 100 mg/ml streptomycin. Third- or fourth-passage cells were used for subsequent experiments.

### Hypoxia treatment

The hypoxia treatment was applied following the protocols described in our previous report [8]. CCSMCs plated in degassing medium were incubated in a modular incubator chamber (StemCell, Vancouver, BC, Canada) filled with hypoxic gas (1% O_2_, 5% CO_2_, and 94% N_2_) and cultured in a 37°C incubator for 24, 48, and 72 h. Normoxic cells were regarded as the control and were incubated under normal conditions (air containing 5% CO_2_).

### RNA interference assay

STAT3 siRNA, PDGFR siRNA, and negative control siRNA were purchased from Sigma (USA). The cells were plated at 50% confluence, transfected with 50 nM siRNA complexed with Lipofectamine 2000 (Invitrogen) in Opti-MEM overnight, and then incubated for various amounts of time.

### STAT3 overexpression in CCSMCs

Lentivirus carrying cDNA for STAT3 and control virus were purchased from Shanghai Genechem Company. CCSMCs were inoculated in 12-well plate and incubated with complete medium containing lentivirus. At 24 h after seeding, the medium containing the lentivirus was aspirated and replaced with 1 ml of fresh medium overnight, and the cells were cultured for 2 days. To obtain a stably transduced cell line, the lentivirus-infected cells were treated with culture medium containing 2 μg/ml puromycin, and the culture medium was changed every three days.

### *In vitro* cell assay

The cells were seeded in 6-well plates at a density of 50,000 cells per well. After 24 h, the cells were treated with PDGF-BB at a concentration of 0, 5, 10, 20 ng/ml or crenolanib at a concentration of 100 nM for 24 h. The cells were then collected for protein extraction.

### Immunofluorescence staining

Immunofluorescence staining was performed to identify cells. The cells were fixed in 4% formaldehyde (freshly prepared from paraformaldehyde) in PBS (pH 7.4) for 30 min and permeabilized for 15 min with 0.5% Triton X-100 in PBS. The CCSMCs were rinsed with PBS, blocked in 10% goat serum for 1 h, and incubated overnight with a primary antibody against α-SMA; the antibody was diluted 1:1000 in skimmed milk [22] (Abcam, Cambridge, MA, USA) and desmin [23] (Santa Cruz Biotechnology, Santa Cruz, CA, USA). The cells were then incubated with a fluorescein isothiocyanate (FITC)-conjugated secondary antibody (Abcam) at 25–30°C for 30 min. Finally, the CCSMCs were analyzed using a Nikon Eclipse Ti inverted microscope (Nikon, Tokyo, Japan).

### Western blotting

CCSMCs were cultured in 6-well plates and treated with PDGF-BB at 5, 10, or 20 ng/ml or PDGFR siRNA for 48 h. The cells were lysed in cell lysis buffer (20 mM Tris [pH 7.5], 150 mM NaCl, 1 mM Na_2_EDTA, 1 mM EGTA, 1% TritonX-100, 2.5 mM sodium pyrophosphate, 1 mM beta-glycerophosphate, 1 mM Na_3_VO_4_, and 1 μg/ml leupeptin) for 30 min on ice and centrifuged at 8,000*g* for 10 min. The protein concentration was determined using a BCA protein assay kit (Pierce, Rockford, IL, USA). Then, 40 μg of cellular protein was loaded onto 10% SDS-PAGE gels and transferred to polyvinylidene fluoride (PVDF) membranes (Millipore Corporation, Bedford, MA, USA). After blocking with Tris buffer solution containing 5% non-fat milk for 1 h at 25–30°C, the membranes were incubated overnight at 4°C with primary antibodies against α-SMA, desmin, vimentin, collagen-I, STAT3, p-STAT3, STAT5, p-STAT5, AKT, p-AKT, ERK, p-ERK, PDGFRα, p-PDGFRα, PDGFRβ, p-PDGFRβ, and β-actin; the antibodies were diluted 1:1000 with skim milk [24–29]. After thorough washing, the blots were incubated with a horseradish peroxidase (HRP)–conjugated secondary antibody diluted 1:1000 in skim milk. Protein band intensity was analyzed using enhanced ECL reagents (Amersham, Piscataway, NJ, USA) and a Versa Doc MP5000 imaging system (Bio-Rad, Hercules, CA, USA).

### Statistical analysis

All experiments were performed in triplicate. Data were analyzed using one-way analysis of variance (ANOVA) followed by a post hoc test in SPSS 15.0 software (SPSS Inc., Chicago, IL, USA). The data have been expressed as the mean ± standard error of mean (SEM). P<0.05 was considered to indicate statistical significance.

## Results

### Isolation and characterization of CCSMCs

**Fig 1:**
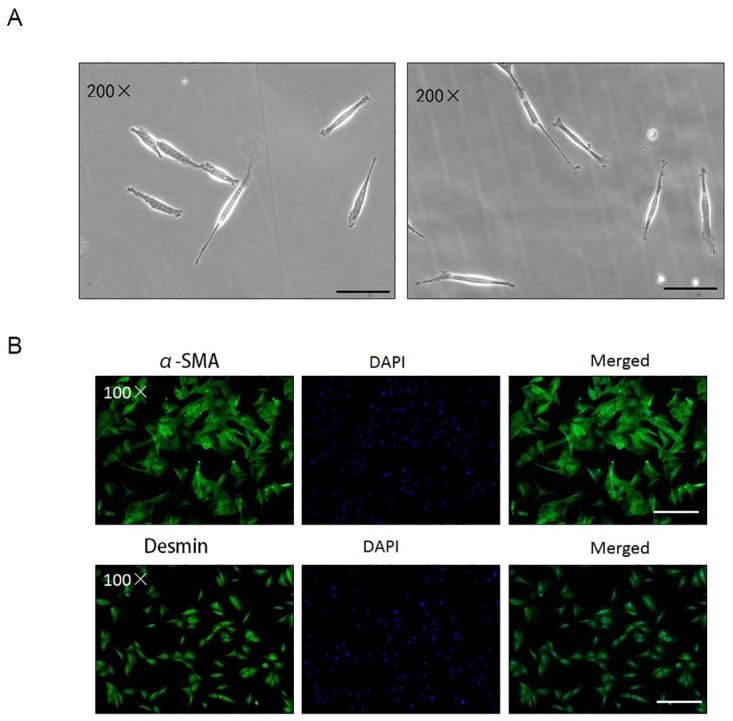
Identification and characterization of primary cultured CCSMCs. Scale bars = 20 μm. (A) Microscopic imaging of first- to second-passage primary cells. (B) Immunostaining images of primary CCSMCs stained with multiple markers. Scale bars = 100 μm.

The primary CCSMCs initially appeared to have a spindle shape and maintained their original morphological features even in the second passage (Fig 1A). Immunofluorescence staining studies showed that the isolated cells predominantly included SMCs that stained positively for α-SMA and desmin (Fig 1B), consistent with a previous report [30].

### Hypoxia induced changes in the expression of proteins associated with CCSMC phenotype and PDGF receptors

**Fig 2:**
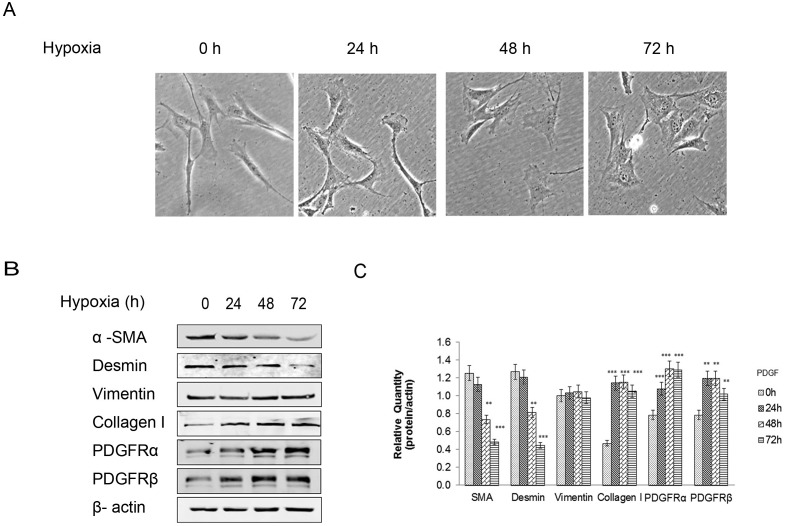
Hypoxia-induced CCSMC phenotype transition and PDGFR protein upregulation. (A) Microscopic imaging of CCSMCs exposed to hypoxia of 0, 24, 48, 72 h. Scale bars = 20 μm. (B,C)Western blot analysis measuring CCSMC phenotypic-related proteins and PDGFR proteins, including α-SMA, desmin, vimentin, collagen-I, PDGFRα, and PDGFRβ. β-Actin was used as the control. Data are the mean tumor volume ± SD. *P*<0.05 was considered statistically significant. * *P*<0.05, ** *P*<0.01, versus normoxic control group (n = 4).

CCSMCs were exposed to hypoxic conditions for 24, 48, and 72 h, and the morphology and expression of phenotype-associated proteins of CCSMCs was analyzed. Hypoxia altered CCSMC morphology from the original elongated, spindle shape to a broader shape (Fig 2A). Hypoxic cells showed significantly decreased expression of α-SMA and desmin and significantly increased expression of vimentin and collagen-I compared with the normoxic control cells. Furthermore, western blotting analysis showed that protein expression of PDGFRα and PDGFRβ was increased in hypoxic CCSMCs. Obvious changes of β-actin protein expression were not observed (Fig 2B and 2C).

### PDGF-BB promoted the CCSMC synthetic phenotype and the expression of related proteins

**Fig 3:**
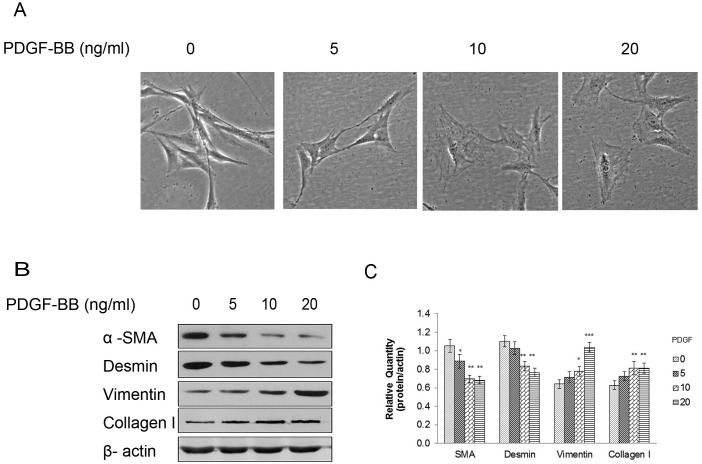
PDGF-BB-induced changes in CCSMC phenotypes. (A) Microscopic imaging of CCSMCs exposed to 0, 5, 10, or 20 ng/ml PDGF-BB. Scale bars = 20 μm. (B, C) Western blot results showing increased vimentin and collagen-I expression and decreased α-SMA and desmin expression on exposure to gradient-elevated PDGF-BB. β-Actin was used as the control. Data are the mean tumor volume ± SD. *P*<0.05 was considered statistically significant. * *P*<0.05, ** *P*<0.01, versus control (n = 4).

CCSMCs were exposed to 5, 10, or 20 ng/ml PDGF-BB; the shape of the cells changed from the original elongated, spindle shape to a broader shape than that noted for normal cells (Fig 3A). To confirm the effect of PDGF on the phenotype of CCSMCs, the expression of CCSMC markers was analyzed. PDGF-BB–treated cells showed considerably reduced α-SMA and desmin expression and increased vimentin and collagen-I expression, whereas β-actin expression did not show obvious changes (Fig 3B).

### Downregulation of PDGFR via PDGFR siRNA and crenolanib inhibited the CCSMC synthetic phenotype and promoted the contractile phenotype

**Fig 4:**
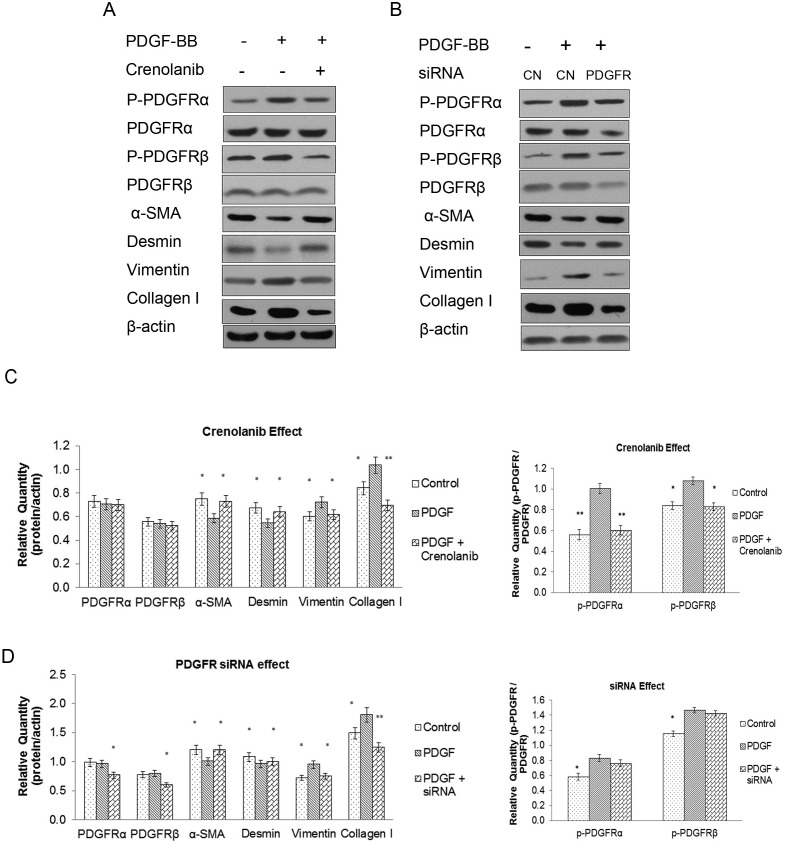
Inhibition of PDGFR activity changed the phenotypic transformation. Western blot analysis of CCSMC phenotype-related proteins, including α-SMA, desmin, vimentin, and collagen-I, after treatment with PDGF-BB at 20 ng/ml, PDGFR siRNA at 50 nM (A, C), or Crenolanib at 100 nM (B, D). Data are the mean tumor volume ± SD. *P* <0.05 was considered statistically significant. * *P*<0.05, ** *P*<0.01 versus PDGF-BB group (n = 4).

To confirm the effect of the PDGFR pathway on the CCSMC phenotype, the cells were treated with PDGFR siRNA or crenolanib, and phenotype-associated proteins were detected. PDGFR siRNA and crenolanib treatment reversed the phenotypic transition induced by PDGF-BB that downregulated the synthetic phenotype–associated proteins vimentin and collagen-I and upregulated the contractile phenotype–associated proteins α-SMA and desmin. In addition, crenolanib reduced the proportion of phosphorylated PDGFR proteins but had no effect on the expression of total proteins; in contrast, PDGFR siRNA decreased the total protein expression but not the ratio of phosphorylation (Fig 4).

### Inhibition of PDGFR activity significantly decreased AKT and STAT3 phosphorylation, and knockdown of STAT3 promoted the contractile phenotype

**Fig 5:**
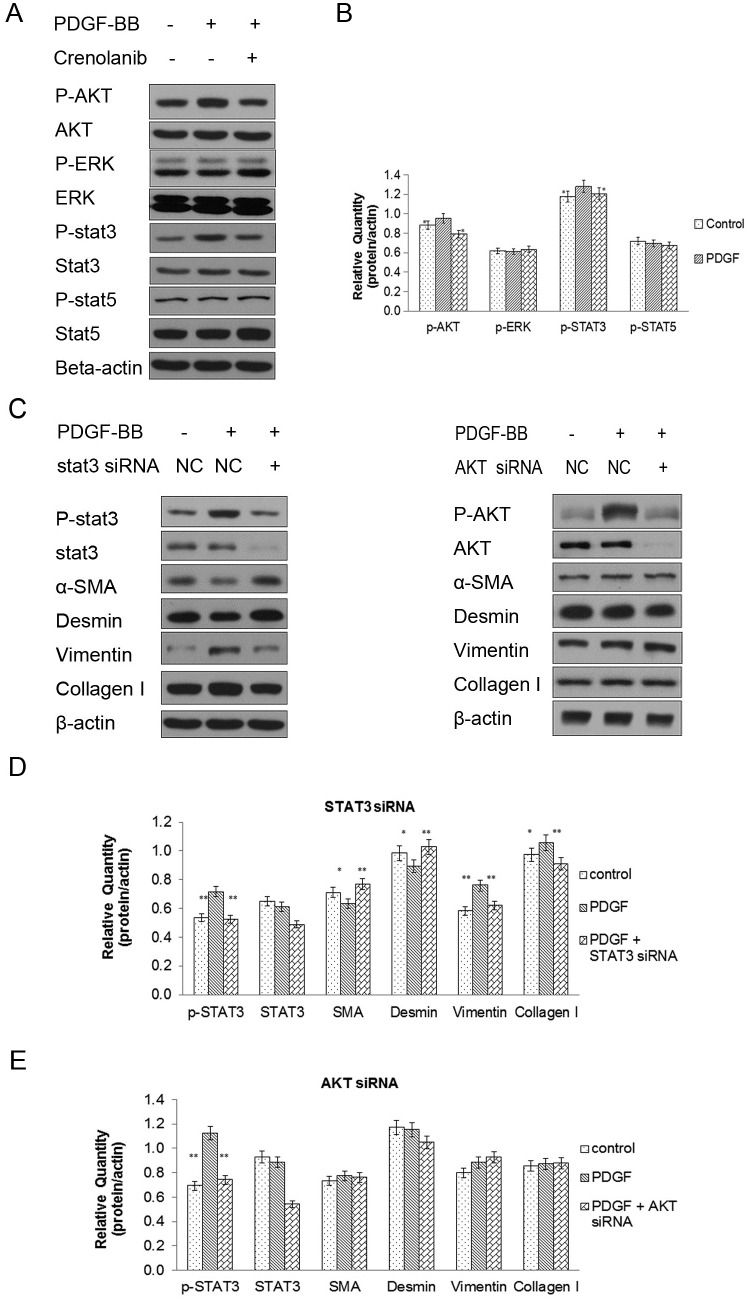
Proteins downstream of PDGFR and associated with changes in the CCSMC phenotype. (A, B) Western blot analysis of proteins downstream of PDGFR after crenolanib treatment, including p-AKT, AKT, p-ERK, ERK, p-STAT3, STAT3, p-STAT5, and STAT5 after treatment with PDGF-BB at 20 ng/ml or crenolanib at 100 nM. (C, D, E) Western blot analysis of CCSMC phenotype-related proteins after treatment with STAT3 or AKT siRNA at 50 nM. β-actin protein was used as the control. Data are the mean tumor volume ± SD. *P*<0.05 was considered statistically significant. * *P*<0.05, ** *P*<0.01, versus control (n = 4).

To identify the regulatory proteins in the CCSMC-associated phenotype from among the PDGFR downstream proteins, vital proteins in the PDGFR pathway were investigated after crenolanib treatment. Western blotting results suggested that inhibition of PDGFR activity significantly inhibited AKT and STAT3 phosphorylation and reduced the proportion of phosphorylated proteins (Fig 5A and 5B). Then, we knocked down STAT3 expression, which promoted the contractile phenotype, downregulated vimentin and collagen-I expression, and upregulated α-SMA and desmin expression. AKT siRNA knockdown did not affect phenotype-associated proteins (Fig 5C–5E).

### STAT3 overexpression in CCSMCs weakened the inhibitory effect of PDGFR on phenotypic transformation

**Fig 6:**
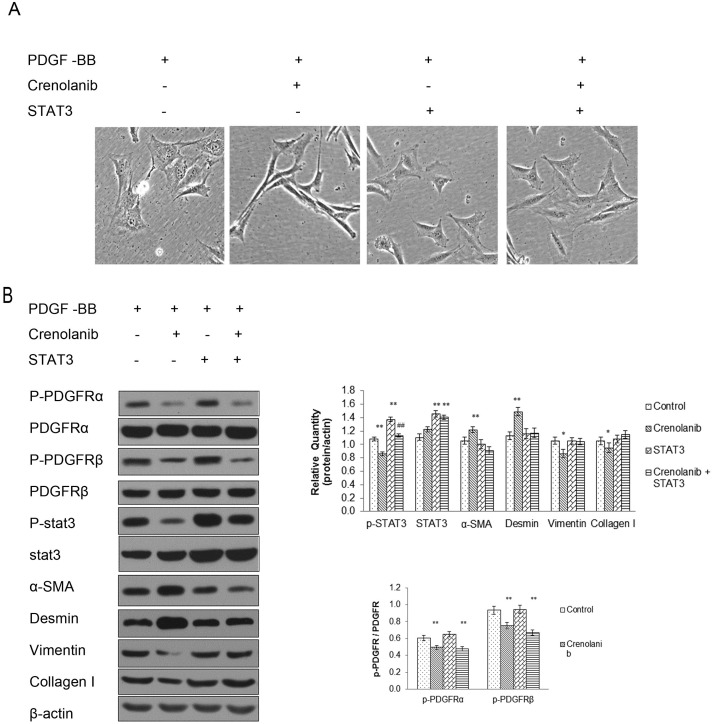
STAT3 overexpression changed the phenotypic transformation. **(A)** Microscopic imaging of CCSMCs exposed to PDGF-BB at 20 ng/ml or Crenolanib at 100 nM, or when STAT3 was overexpressed. Scale bars = 20 μm. **(B)** Western blot analysis of phenotype-related proteins. The expression of these proteins showed alterations after treatment with PDGF-BB at 20 ng/ml or Crenolanib at 100 nM, or when STAT3 was overexpressed in CCSMCs. Data are the mean tumor volume ± SD. *P*<0.05 was considered statistically significant. * *P*<0.05, ** *P*<0.01 versus control group; # *P*<0.05, ## *P*<0.01 versus STAT3 overexpression group (n = 4).

We further investigated the role of STAT3 in the CCSMC phenotype. Crenolanib inhibited the morphological changes from spindle shape to broader shape induced by PDGF-BB, but STAT3 overexpression could weakened the effect of crenolanib (Fig 6A). Western blot results also showed synthetic phenotype induced by PDGF-BB via downregulation of vimentin and collagen-I expression and upregulation of α-SMA and desmin expression. However, when STAT3 was overexpressed in CCSMCs, the effect of crenolanib on phenotypic transformation was significantly weakened (Fig 6B and 6C), which indicates that PDGF affects the phenotype of CCSMCs via the PDGFR/STAT3 pathway. Partial original data set see supporting information S1 File and S1 Table.

## Discussion

Smooth muscle damage is an important cause of ED and may lead to decreased [22,31] or increased SMC content [32]. α-SMA and desmin have been accepted as differentiation markers for SMCs [33]. Our results showed that the isolated cells mainly included CCSMCs with the contractile phenotype. Cellular morphology is another important marker of SMC behavior [34,35]. *In vitro*, CCSMCs in the contractile state exhibit spindle or rhomboid morphology, whereas CCSMCs in the synthetic state appear broader with a larger diameter [36,37]. Phenotype modulation of SMCs has been involved in many human diseases, including atherosclerosis and restenosis, which are believed to be caused by early phenotypic changes from the contractile to synthetic phenotype in vascular SMCs in arteries [38,39].

PDGF secreted by senescent cells is a growth factor that regulates cell growth and division of pericytes, which cover endothelial cell channels to provide stability and control perfusion of blood vessels [40]. PDGF is a key mediator of vascular SMC phenotypic modulation [41]. PDGFR is a transmembrane receptor tyrosine kinase activated by PDGF. After activation, tyrosine phosphorylation of the receptor activates several downstream signaling pathways, including the phosphatidylinositol 3-kinase/serine-threonine protein kinase (PI3K/AKT), ERK1/2, phospholipase C/protein kinase C (PLC/PKC), and STAT pathways, which are involved in processes such as cell growth and survival, transformation, and migration [42,43].

Incubation under hypoxic conditions stimulates PDGF and PDGFR expression in the rat corpus cavernosum *in vivo* [44]. Furthermore, in impotent penile tissue in the case of Peronei’s disease and venocclusive dysfunction, fibroblasts from tunica albuginea tissue with pathological changes show increased PDGF expression [19]. Consequently, higher PDGF and PDGFR expression may determine imbalance in CCSMCs, resulting in ED. In this study, *in vitro* analysis showed morphological and phenotypic changes and PDGFR upregulation in CCSMCs subjected to hypoxic conditions. Moreover, CCSMCs also underwent changes from a contractile to a synthetic phenotype when treated with PDGF-BB, which was associated with significantly decreased α-SMA and desmin expression and increased vimentin and collagen-I expression. We also found similar changes in their morphology from the original elongated, spindle shape to a broader shape, which constitutes the initial stage of fibrosis. When the cells were treated with PDGFR siRNA or the PDGFR inhibitor crenolanib, the CCSMC synthesis phenotype was induced by PDGF-BB with decreased vimentin and collagen-I expression and promotion of the contractile phenotype with increasing α-SMA and desmin expression.

In the last few years, a latent relationship between the ERK, AKT, and STAT signaling pathways and phenotype modulation has been found [45–47]. In the present study, we found that inhibition of PDGFR activity significantly inhibited AKT and STAT3 phosphorylation, which indicates that PDGFR inhibition regulates the CCSMC synthesis phenotype via phosphorylation of proteins downstream of PDGFR. We knocked down STAT3 expression and found that it promoted the contractile phenotype, inhibited vimentin and collagen-I expression, and promoted α-SMA and desmin expression. However, knockdown of AKT did not affect the phenotype-associated proteins. STAT3 is a transcription factor that is activated primarily by tyrosine phosphorylation and then transcriptionally modulates a range of targeted genes. A possible link between STAT3 signaling and phenotype modulation was suggested by a previous study [48]. In that study, STAT3 knockdown was found to decrease the phosphorylation of STAT3 and enhance the vascular SMC contractile phenotype by interaction with myocardin. In the present study, we observed that STAT3 overexpression in CCSMC cells weakened the effect of the PDGFR inhibitor on phenotypic transition induced by PDGF-BB. Thus, we suggest that the PDGFR/STAT3 signaling pathway regulates the phenotypic transition of CCSMCs and that STAT3 phosphorylation plays a vital role in the PDGFR/STAT3 signaling pathway.

In conclusion, PDGF induced phenotype modulation of primary CCSMCs through activation of the PDGFR/STAT3 signaling pathway. It is conceivable that this pathway may be involved in the pathogenesis of ED, which frequently complicates atherosclerosis and deregulates the PDGFR/STAT3 pathway, and might contribute to clinical ED therapy. The *in vivo* effect of PDGF requires further study.

## Supporting information

S1 File The original, uncropped and unadjusted blots generated for proteins. (RAR)

S1 Supporting Information Underlying dataset. (RAR)

S1 Table The original data for protein quantification. (XLSX)
